# Evolutionary loss of melanogenesis in the tunicate *Molgula occulta*

**DOI:** 10.1186/s13227-017-0074-x

**Published:** 2017-07-18

**Authors:** Claudia Racioppi, Maria Carmen Valoroso, Ugo Coppola, Elijah K. Lowe, C. Titus Brown, Billie J. Swalla, Lionel Christiaen, Alberto Stolfi, Filomena Ristoratore

**Affiliations:** 1 Biology and Evolution of Marine organisms, Stazione Zoologica Anton Dohrn, Villa Comunale, 80121 Naples, Italy; 2 Center for Developmental Genetics, Department of Biology, New York University, New York, NY USA; 3 Station Biologique de Roscoff, Roscoff, France; 4 Department of Biology, University of Washington, Seattle, WA USA; 5 Friday Harbor Laboratories, University of Washington, Friday Harbor, WA USA; 6 Population Health and Reproduction, UC Davis School of Veterinary Medicine, Davis, CA USA; 7 School of Biological Sciences, Georgia Institute of Technology, Atlanta, GA USA; 8 Department of Biology, University of Naples Federico II, Naples, Italy

**Keywords:** Pigmentation, Tyrosinase evolution, Transposable elements, Pseudogenes, Phylogeny

## Abstract

**Background:**

Analyzing close species with diverse developmental modes is instrumental for investigating the evolutionary significance of physiological, anatomical and behavioral features at a molecular level. Many examples of trait loss are known in metazoan populations living in dark environments. Tunicates are the closest living relatives of vertebrates and typically present a lifecycle with distinct motile larval and sessile adult stages. The nervous system of the motile larva contains melanized cells associated with geotactic and light-sensing organs. It has been suggested that these are homologous to vertebrate neural crest-derived melanocytes. Probably due to ecological adaptation to distinct habitats, several species of tunicates in the Molgulidae family have tailless (anural) larvae that fail to develop sensory organ-associated melanocytes. Here we studied the evolution of *Tyrosinase* family genes, indispensible for melanogenesis, in the anural, unpigmented *Molgula occulta* and in the tailed, pigmented *Molgula oculata* by using phylogenetic, developmental and molecular approaches.

**Results:**

We performed an evolutionary reconstruction of the tunicate *Tyrosinase* gene family: in particular, we found that *M. oculata* possesses genes predicted to encode one Tyrosinase (Tyr) and three Tyrosinase-related proteins (Tyrps) while *M. occulta* has only *Tyr* and *Tyrp.a* pseudogenes that are not likely to encode functional proteins. Analysis of *Tyr* sequences from various *M. occulta* individuals indicates that different alleles independently acquired frameshifting short indels and/or larger mobile genetic element insertions, resulting in pseudogenization of the *Tyr* locus. In *M. oculata, Tyr* is expressed in presumptive pigment cell precursors as in the model tunicate *Ciona robusta*. Furthermore, a *M. oculata Tyr* reporter gene construct was active in the pigment cell precursors of *C. robusta* embryos, hinting at conservation of the regulatory network underlying *Tyr* expression in tunicates. In contrast, we did not observe any expression of the *Tyr* pseudogene in *M. occulta* embryos. Similarly, *M. occulta Tyr* allele expression was not rescued in pigmented interspecific *M. occulta × M. oculata* hybrid embryos, suggesting deleterious mutations also to its *cis*-regulatory sequences. However, in situ hybridization for transcripts from the *M. occulta Tyrp.a* pseudogene revealed its expression in vestigial pigment cell precursors in this species.

**Conclusions:**

We reveal a complex evolutionary history of the melanogenesis pathway in tunicates, characterized by distinct gene duplication and loss events. Our expression and molecular data support a tight correlation between pseudogenization of *Tyrosinase* family members and the absence of pigmentation in the immotile larvae of *M. occulta*. These results suggest that relaxation of purifying selection has resulted in the loss of sensory organ-associated melanocytes and core genes in the melanogenesis biosynthetic pathway in *M. occulta.*

**Electronic supplementary material:**

The online version of this article (doi:10.1186/s13227-017-0074-x) contains supplementary material, which is available to authorized users.

## Background

Closely related species with different modes of development are attractive systems to investigate the molecular bases of evolutionary changes. The evolutionary loss of physiological, anatomical and behavioral traits by cave-dwelling animals is one noteworthy example [1]. For instance, several studies have revealed convergent or parallel evolution of loss of pigmentation, eyes and various behavioral adaptations in various populations of the Mexican cave tetra (*Astyanax mexicanus*), which have evolved in parallel from surface-dwelling populations [2–4]. Insights into similar processes in cave-dwelling crustaceans are beginning to emerge [5].

Tunicates are the sister group to the vertebrates and are characterized by a life cycle with distinct motile larval and sessile adult stages [6, 7]. The tailed, tadpole-type larva of a typical tunicate consists of a head containing a brain, and a tail containing a dorsal nerve cord and a notochord flanked by striated muscle cells. Larvae swim by alternated left–right contractions of the tail muscles, which are controlled by a minimal central nervous system containing only 177 neurons [8]. Inside the brain, two sensory organs containing a single melanocyte each are generally present: the otolith, for gravity perception, and the ocellus, for the detection of light [9, 10]. These two distinct pigment cells develop from a single bilateral pair of cells along the neural plate borders [11] and are proposed to be homologous to pigment cells of the dorsal ocelli in amphioxus [12, 13] and to neural crest-derived melanocytes in vertebrates [14, 15]. Pigmentation in tunicates depends critically on the activity of melanogenic enzymes Tyrosinase (Tyr) and Tyrosinase-related proteins (Tyrps) [16, 17], as is the case in vertebrates [18].

Despite the deeply conserved body plan of the swimming tunicate larva, some species instead develop a tailless (anural) larva which is immotile [19–21]. About 20 tunicate species have been shown to exhibit anural development, and almost all of these species are members of the family Molgulidae, in which tail loss has occurred independently multiple times [21, 22]. The ecological significance of these losses is still not clear, although it has been suggested that this could be related to the habitats to which these species are adapted, notably sand flats and rocky shores at higher latitudes [22].

Anural tunicate larvae have conspicuously lost most of those features more directly associated with swimming. For instance, they have lost the ability to generate differentiated tail muscles [23–28], and produce rounded larvae without the characteristic elongated, striated tail muscles typical of most tunicate larvae. The anural species *Molgula occulta* has lost genes important for muscle function such as *Muscle actin,* which has become pseudogenized by deletions, insertions and codon substitutions in coding and noncoding regions, resulting in truncated proteins and/or reduced transcriptional activity [29, 30]. However, tail muscles are still specified in anural embryos, which express the same regulatory cascade for myogenesis in tailed species [31, 32]. Similarly, anural embryos also specify notochord cells, but these later fail to undergo proper morphogenesis by convergent extension [33].

In contrast the loss of functional muscles and notochord, the loss of differentiated neural structures in anural larvae has not been studied in detail. The most obvious evolutionary loss of a neural structure is that of the sensory pigment cells, or melanocytes. While tunicate larvae generally possess both an ocellus and otolith, most molgulids have only an otolith. They have lost the ocellus and, concomitantly, the ability to respond to light [34]. In *Molgula* species with anural larvae, there has been a more complete loss of pigmentation, as even the otolith is missing [19] or vestigial [26]. This has been postulated to be due the lack of adaptive value of the otolith for such anural larvae, which do not swim and therefore do not need to sense their orientation in the water column as swimming, tailed larvae do [22].

Here we describe the development of sensory pigment cells in the embryos of *Molgula oculata,* and the molecular basis of pigmentation loss in the closely related, anural and unpigmented species *M. occulta*. We found that inactivated *Tyrosinase* (*Tyr*) and *Tyrosinase-related protein* (*Tyrp*) genes are present in the genome of *M. occulta,* but are not likely to produce functional enzymes. While *Tyr* transcripts were not detected in *M. occulta* embryos, transcription of a *Tyrp* gene was observed in vestigial pigment cells. Surprisingly, more than two distinct *Tyrosinase* alleles with different inactivating mutations were cloned from a sampling of *M. occulta* individuals, suggesting parallel loss of pigmentation by independent loss-of-function mutations *Tyrosinase* within the same population.

## Results

### Tyrosinase family enzymes in *Molgula*

The melanogenic toolkit of tunicates and vertebrates revolves around *Tyrosinase* family gene members, which encode the rate-limiting enzymes that catalyze the final steps in melanin biosynthesis [16]. In *Ciona* spp., the family is comprised by *Tyrosinase* (*Tyr*)*, Tyrosinase-related protein a* (*Tyrp.a*) and *Tyrosinase-related protein b* (*Tyrp.b*). We searched for orthologs of these genes by performing tBlastn [35] on the recently published genomes of tailless, unpigmented species *Molgula occulta* and the closely related, tailed, pigmented species *Molgula oculata* [36], using the *Ciona robusta* (formerly known as *Ciona intestinalis* type A) [37] and *Halocynthia roretzi* [38] orthologs as query sequences.

**Fig. 1:**
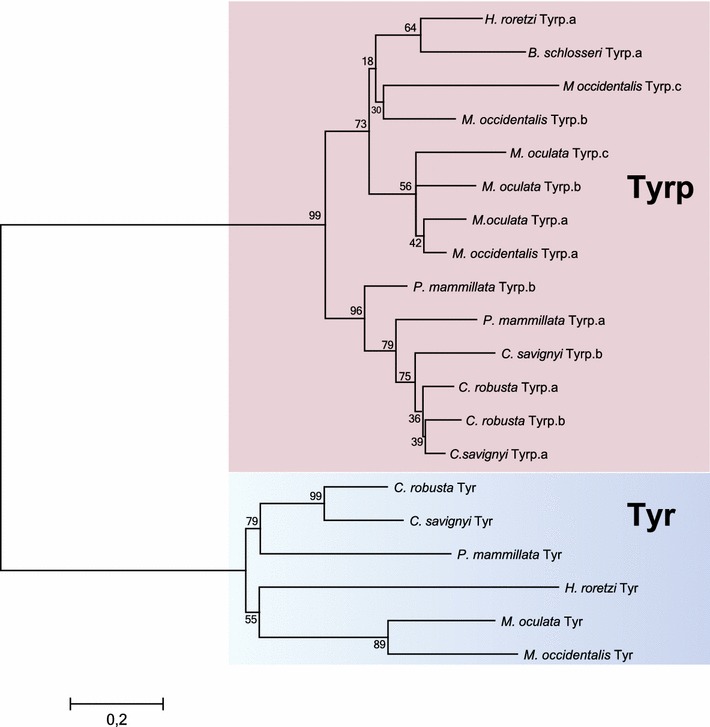
Tyrosinase family evolution in tunicates. Maximum likelihood phylogeny of Tyrosinase members available in databases: The numbers at the branches represent the replicates come out employing ML method. Two distinct *colored boxes* have been used to highlight Tyr (*blue box*) and Tyrp (*pink box*) protein classes

We found partial sequences for one *Tyr* (*Mooccu.Tyr*) and one *Tyrp* (*Mooccu.Tyrp.a*) ortholog in the genome of *M. occulta.* In contrast, we were able to identify a single *Tyr* ortholog (*Moocul.Tyr*) and at least four distinct *Tyrp* paralogs in the genome of the *M. oculata* (*Moocul.Tyrp.a, Moocul.Tyrp.b, Moocul.Tyrp.c* and *Moocul*.*Tyrp.d*). Previously, phylogenetic analyses had shown that *Ciona Tyrp.a* and *Tyrp.b* are the result of a tunicate-specific independent duplication of an ancestral *Tyrp* gene, equally related to the vertebrate paralogs *Tyrp1* and *Tyrp2* (*Dopachrome tautomerase*) [16]. An ascidian phylogenetic tree encompassing *C. robusta*, *Ciona savignyi* and *Phallusia mammillata* for Phlebobranchia, *Halocynthia roretzi*, *Botryllus schlosseri*, *M. occulta*, *M. oculata* and *M. occidentalis* for Stolidobranchia sequences does not support one-to-one orthology between any of the *Ciona* and *Molgula Tyrp* genes (Fig. 1), suggesting that a single ancestral *Tyrp* gene likely underwent independent duplications in different tunicate clades.

We also searched for *Tyr* and *Tyrp* mRNA transcripts in the transcriptomes obtained from embryos of both species at three different developmental stages (late gastrula, neurula and tailbud) [39]. Sequences from all *Tyr* family genes from *M. oculata* were identified in the corresponding transcriptome, suggesting that all these genes are expressed during embryogenesis of pigmented *Molgula* species. By contrast, only *Mooccu.Tyrp.a* was present in the *M. occulta* embryonic transcriptome, hinting at the possibility that the melanogenic toolkit is only partially deployed during the development of this species’ unpigmented, anural larvae. Furthermore, *Mooccu.Tyr* transcripts were undetectable by RNAseq profiling of interspecies hybrid embryos, resulting from fertilization of *M. oculata* eggs with *M. occulta* sperm [39]. While 70–215 RNAseq reads could be mapped to the *M. oculata Tyr* allele in hybrid embryos at each developmental stage, only one or two reads mapped to *M. occulta Tyr* alleles at any stage (see Table in Additional file 1). Thus, even in embryos actively transcribing the *M. oculata Tyr* allele at high levels, the *M. occulta* allele is largely transcriptionally inactive. Taken together, these data indicate that the *M. occulta Tyr* gene is transcriptionally silent during embryonic development. Since hybrid embryos have pigmented cells [33], our data also indicate that this rescue of melanization in the interspecific hybrids is due entirely to the *M. oculata Tyrosinase* allele.

### Pigment cell marker expression in *Molgula* embryos

**Fig. 2:**
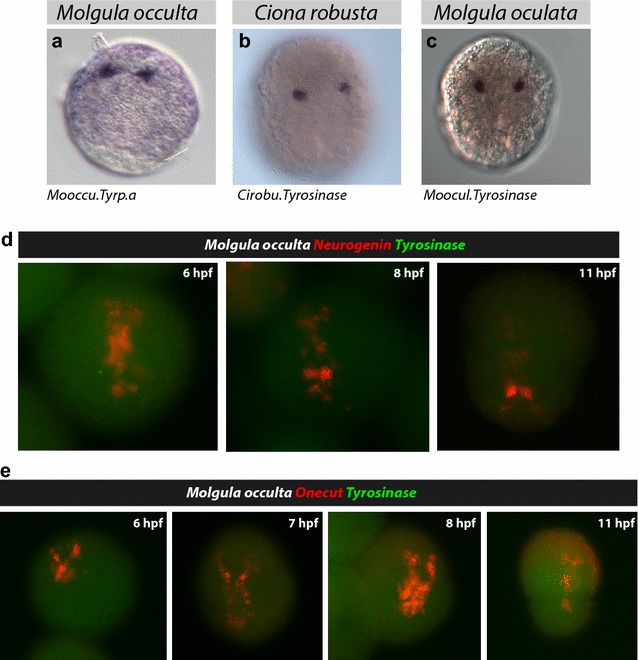
Expression of *Tyrosinase* genes during tunicate embryogenesis. The whole-mount in situ hybridization experiments show the localization of *Tyrosinase* gene expression in pigment cell precursors of three tunicates: Mooccu.Tyrp.a in *Molgula occulta* hatched larva (**a**), *Cirobu.Tyr* in *Ciona robusta* neurula (**b**), *Moocul.Tyr* in *Molgula oculata* neurula embryos (**c**). Double in situ hybridization of *M. occulta Tyrosinase* and either *Neurogenin* (**d**) or *Onecut* (**e**), showing lack of *Tyrosinase* expression in the developing central nervous system of embryos and larvae at various stages

We next sought to determine the spatiotemporal expression pattern of selected *M. occulta* and *M. oculata Tyrosinase* family genes, by whole-mount mRNA in situ hybridization on embryos at different developmental stages (Fig. 2). In the larvae of *M. occulta,* bilateral *Mooccu.Tyrp.a* expression was detected in embryos starting at 10 h post-fertilization (hpf), which is around the time they are hatching (Fig. 2a). Their number and location in the embryo suggest they correspond to vestigial pigment cell precursors (a9.49 lineage in *Ciona robusta*, Fig. 2b). In contrast, *Mooccu.Tyr* is absent from the transcriptome (see above) [39]. Indeed, mRNA in situ hybridization using a riboprobe prepared from a genomic clone of the *Mooccu.Tyr* locus (see below) showed no transcriptional activity of this sequence during development nor in hatched larvae (Fig. 2d, e). This further confirms the complete loss of transcriptional activity of this gene in *M. occulta* as suggested by the RNAseq data. Taken together, these data suggest that, although unpigmented *M. occulta* specify vestigial pigment cell precursors that transcribe *Mooccu.Tyrp.a,* the rate-limiting melanogenic gene *Mooccu.Tyr* is not expressed in these cells.

**Fig. 3:**
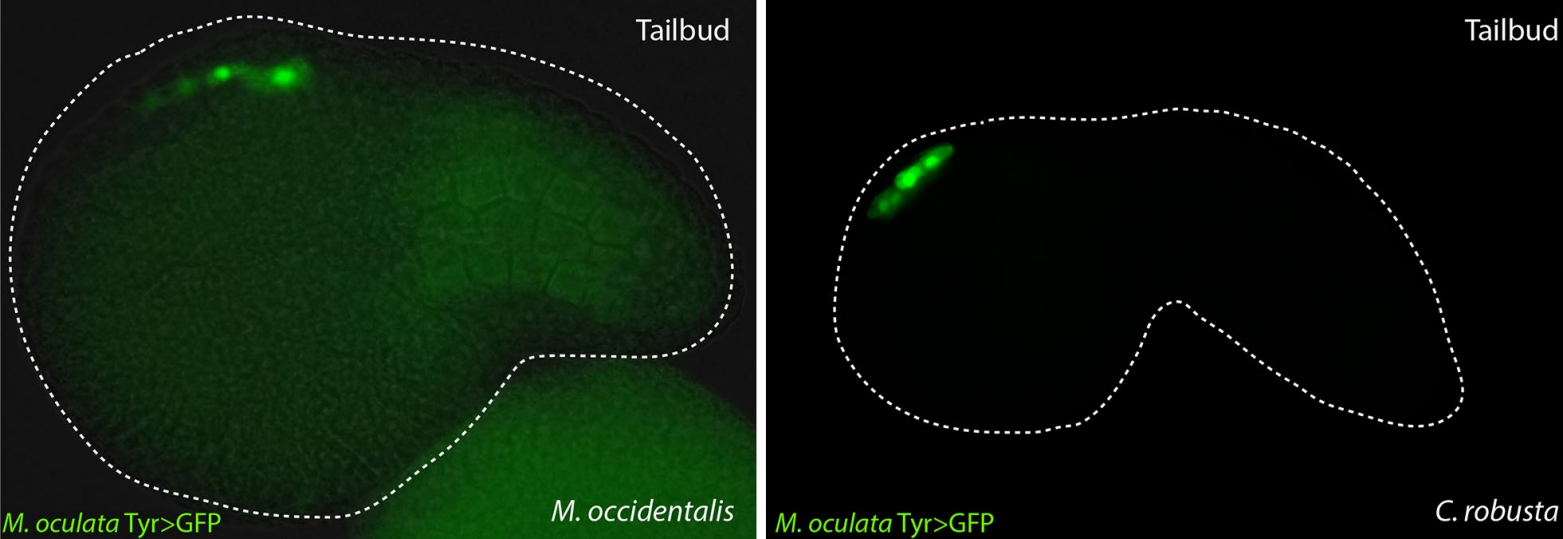
GFP reporter assay of *Molgula oculata Tyr* promoter. A plasmid containing GFP has been used to demonstrate the conservation of *Moocul.Tyr* regulatory activity (1.3 Kb fragment) by electroporation of tunicate embryos. GFP expression is detected in the pigment cell lineage of tailbud-stage embryos of *Molgula occidentalis* (*left*) and *Ciona robusta* (*right*)

To verify whether the *Tyrosinase* ortholog is expressed in the a9.49 lineage cells of the pigmented species *M. oculata,* we performed in situ hybridization on embryos of this species. At 7 hpf, corresponding to neurula stage, *Moocul.Tyr* is expressed in two cells in the neural plate borders corresponding to the pigment cell precursors (Fig. 2c). Later in development at 10 hpf, *Moocul.Tyr* is expressed around the melanizing otolith (see Figure in Additional file 1). We also isolated an intergenic sequence of ~1.3 kbp upstream of the coding ATG in the *Moocul.Tyr* gene and cloned this region in a reporter construct containing GFP (*Moocul.Tyr* > *GFP*). This reporter was tested by electroporation into the pigmented embryos of the distantly related *Molgula occidentalis,* the only *Molgula* species that can be easily transfected with plasmid DNA, and in *C. robusta*. In both species, GFP expression was detected in four cells corresponding to the putative pigment cell lineage in the central nervous system of the tailbud embryo (Fig. 3).

The four cells seen in transgenic *Ciona* and *M. occidentalis* embryos expressing *M. oculata Tyr* > *GFP* reporter are the presumptive ocellus and otolith pigment cells along with their more anterior sister cells that express *Tyr* transiently [40], all converged to form a single row at the midline of the embryo. As mentioned previously, tailed *Molgula* species like *M. oculata* and *M. occidentalis* lack an ocellus and only produce an otolith pigment cell. However, from these data we conclude that in *Molgula* and *Ciona,* both presumptive ocellus and otolith pigment cells are specified early and express *Tyr/Tyrp.a.* Thus, the gene regulatory network for pigment cell specification and *Tyr* activation appears to be conserved between *Molgula* and *Ciona*, but ocellus differentiation in *Molgula* is somehow suppressed after pigment cell specification.

### *Tyrosinase* family pseudogenes in *M. occulta*

The lack of melanin production in *M. occulta* pigment cell lineage hinted at the possibility that one or more *Tyrosinase* family genes have become inactivated in this species, through mutations to coding and/or noncoding sequences. While *Mooccu.Tyr* does not appear to be expressed, transcripts for *Mooccu.Tyrp.a* are still detected. However, this does not necessarily indicate the presence of functional Tyrp.a protein. A precedent for this has been found in the *Muscle actin* genes of *M. occulta,* which have been inactivated by mutations that affect both their *cis*-regulatory and protein-coding sequences [30].

**Fig. 4:**
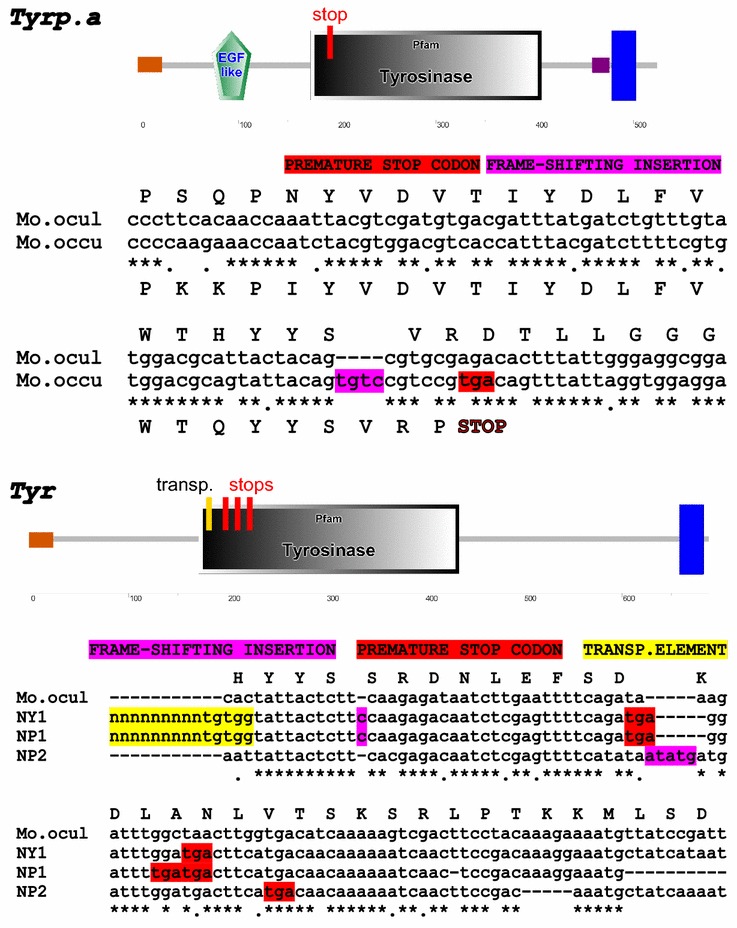
*Tyrosinase* family pseudogenization in *Molgula occulta*. The figure points out the presence of premature stop codons (*red*) and frameshift insertions (*magenta*) in genomic sequences of *Tyrp.a* (**a**) and *Tyr* (**b**). A reconstruction of the active site of the proteins has been obtained using the SMART Web site [58]. Certain *Mooccu.Tyr* alleles are characterized by the presence of transposable elements (*yellow*) inserted just upstream of the active site (**b**)

The alignment of the *M. occulta* and *M. oculata Tyrp.a* sequences from the assembled genomes [36] revealed the presence of a 4-bp insertion in *Mooccu.Tyrp.a*, resulting in a frameshift and a premature stop codon in the middle of the gene, right at the start of the conserved Tyrosinase domain which contains the active site of the enzyme (Fig. 4a). This indicates that *Mooccu.Tyrp.a* transcripts do not code for a full-length, functional enzyme.

We also identified a scaffold from the available genome assembly that includes part of the *Mooccu.Tyr* gene, with an assembly gap in the middle of the protein-coding region. To circumvent this assembly problem and analyze the *Mooccu.Tyr* gene in greater detail, we sought to clone this sequence from genomic DNA extracted from several individuals. To this end, we extracted the genomic DNA from ten individuals and amplified the region from each sample by PCR. This resulted unexpectedly in some individuals carrying *Tyr* alleles of different lengths, suggesting the presence of one or more structural variants of the same *Tyr* gene in the population. Each unique PCR product was purified and cloned into plasmids. When the resulting clones were sequenced, we identified three distinct *Mooccu.Tyr* alleles (Fig. 4b). In the portion of the coding sequence analyzed, one of the alleles (NP2) has a 5-bp frameshifting insertion that results in a premature stop codon, in the beginning of the conserved Tyrosinase domain and enzyme active site (Fig. 4b). The two other alleles (NP1 and NY1) each have a large, ~460-bp insertions with terminal inverted repeats, bearing the hallmarks of a transposable element. These large insertions have several in-frame stop codons. Curiously, NP1 and NY1 have the same element, but in opposite orientations, suggesting one configuration might be derived from the other by a re-transposition event (see Additional file 2). NP1 and NY1 also have a shorter frameshifting insertion downstream of the transposable element, also in the beginning of the Tyrosinase domain, distinct from that of NP2, suggesting a possible independent origin from the functional common ancestor (Fig. 4b).

## Discussion

Melanocytes and related pigment cells share some basic characteristics: Melanin pigments are synthesized by Tyrosinase and Tyrosinase-related enzymes [41] and stored in melanosomes, which are tissue-specific lysosome-related organelles distributed in the cell by a regulated vesicular trafficking system. Loss of pigmentation in humans and other vertebrates can be caused by mutations in *Tyrosinase* itself, or other genes encoding proteins important for substrate and/or pigment transport and storage [42, 43]. Our phylogenetic survey pointed at a complex evolutionary history for Tyrosinase family in ascidians, with a pattern influenced by duplications and losses (Fig. 1). In particular, we discovered a different number of Tyrp paralogs in *Molgula* spp., suggesting the occurrence of many duplicative events in distinct tunicate clades followed, in some cases, by gene losses. It would be interesting to comprehend the evolutionary and functional relevance of these genomic events. It has been recently showed that tunicate melanocyte specification depends on the FGF/MAPK/Ets pathway, which renders the lineage competent to respond to a Wnt signal [44]. Characterization of the transcriptional response to FGF signaling in the pigment cell lineage has allowed us to identify its molecular mechanisms underlying pigment cell fate choice and, more broadly, anterior–posterior patterning of *Ciona* central nervous system [40]. It will be interesting to assess how these events are regulated in *Molgula* species, given that only one pigment cell (the otolith) differentiates.

It was previously suggested that Tyrosinase activity is present in *M. occulta* juveniles, in spite of a lack of discernible pigmentation in juveniles or adults [45]. Here we show that genes coding for key enzymatic components of the melanogenesis pathway, namely *Tyrosinase* and *Tyrp.a*, have been mutated in *M. occulta* and are highly unlikely to code for functional enzymes, calling into question how Tyrosinase enzyme activity could be maintained in this species. Furthermore, assembled genome sequences of three *Molgula* species have not uncovered any cryptic *Tyr* paralogs that could compensate for these loss-of-function mutations. In *Ciona,* a single *Tyr* gene is necessary for melanogenesis and sensory organs are unpigmented in larvae carrying *Tyr* loss-of-function mutations [17]. Our results suggest that the loss of this melanogenic toolkit underlies lack of pigmentation in *M. occulta* and contradicts the alternate hypothesis that the toolkit is present, but the pigment cells are not specified [45].

The identification of three distinct, inactivated alleles of *Mooccu.Tyr* in such a small sample of individuals from the same population suggests pigmentation loss in this species has occurred through accumulation of deleterious mutations, which would indicate a relaxation of the purifying selection that maintains a functional melanogenesis toolkit in species with swimming larvae. This agrees with the suggestion that the ability to detect larval orientation by means of the pigmented otolith is of little adaptive value to the immotile larvae of *M. occulta* [22]. Since melanin appears to be present only in the otolith,  and no other larval or adult tissue, the melanogenesis toolkit would have been dispensable for species with anural larvae such as *M. occulta.*

**Fig. 5:**
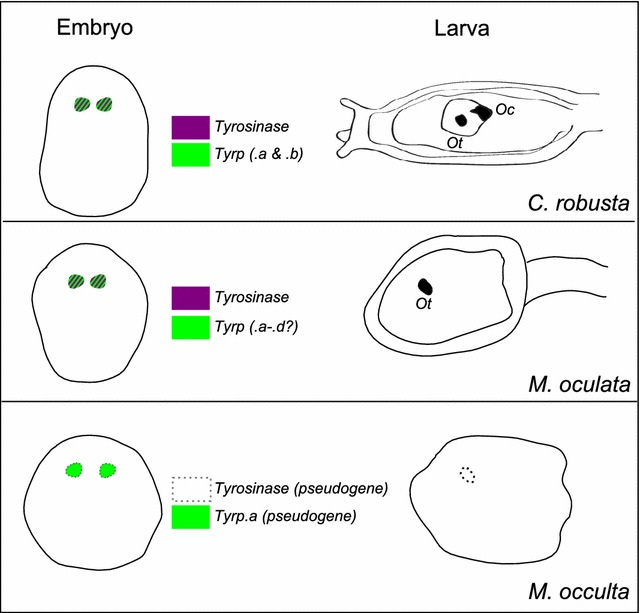
Evolution of pigmentation in tunicates. The scheme correlates the presence or absence of pigmentation in tunicate embryogenesis with the conservation of melanogenic toolkit genes in *Ciona robusta*, *Molgula oculata* and *Molgula occulta*

Furthermore, *Mooccu.Tyrp.a* is predicted to code for a truncated protein, but is still transcribed in the pigment cell lineage, indicating that it still retains functional *cis*-regulatory sequences. This is in contrast to *Mooccu.Tyr,* which is not expressed (even in interspecific hybrids) and thus is likely to bear inactivating mutations in its *cis*-regulatory sequences as well (Fig. 5). This diversity of mutations suggests that loss of melanogenesis pathway genes is still an ongoing process in *M. occulta* populations. Loss of pigmentation in different populations of *Astyanax* cavefish can occur through independent mutations in the same gene [4]. In contrast, loss of pigmentation in the cave crustacean *Asellus aquaticus* can occur through mutations in different genes in the same population [5]. Our discovery of pigment loss in *M. occulta* through independent mutations in the *same* gene in the *same* population is thus a distinct variation on this theme. It will be interesting to understand how population structure and the genetic architecture of pigmentation in *Molgula* may have contributed to this.

## Conclusions

This study shed light on the molecular mechanisms underlying loss of pigmentation in the annual larvae of *Molgula occulta* (Fig. 5). Our data point to a role of independent mutations in at least two *Tyrosinase* family genes in the same population. These mutations are highly likely to result in enzymatically inactive Tyr and Tyrp proteins, which would explain pigment loss in *M. occulta*, even though we show this species still specifies pigment cell precursors like its pigmented relatives. The identification of several seemingly independent loss-of-function *Tyr* mutations in the *M. occulta* population suggests a relaxation of purifying selection may have contributed to loss of pigment cells in this species.

## Methods

### Phylogenetic analysis

In phylogenetic reconstruction, we employed sequences that have been retrieved from the NCBI (http://www.ncbi.nlm.nih.gov), Ensembl (http://www.ensembl.org/index.html) and Aniseed (http://www.aniseed.cnrs.fr/) [46] databases excluding uninformative proteins (see Additional file 3). *Ciona robusta* Tyr and Tyrp.a were the initial query sequences used for TBLASTN [35] in other tunicates, and reciprocal blasts were performed on every genome/transcriptome [36, 46, 47]. The protein set was aligned by ClustalW with default parameters [48]. The phylogenetic tree inference was computed employing the maximum likelihood estimation (MLE) using MEGA6 with the WAG + γ + I matrix [49], in order to improve sensitiveness in our analysis. We assessed the robustness of tree topologies with 1000 bootstrap replicates, and the numbers at the branches indicate replicates obtained using the maximum likelihood estimation method (Fig. 1). The graphical representation of trees was constructed using Dendroscope [50].

### *M. occulta × M. oculata* hybrid embryo transcriptome analysis

Reads from RNAseq of *M. occulta × M. oculata* hybrid embryos from three different stages, gastrula, neurula and tailbud [39], were mapped to the three *M. occulta Tyr* allele sequences cloned, or the *M. oculata Tyr* transcript sequence, using Salmon v0.6.0 [51].

### Molecular cloning

Putative orthologs of *Ciona robusta* and *Halocynthia roretzi* protein-coding genes were initially identified by TBLASTN. Identified sequences were aligned to each other to support orthology relationships within *Molgula* and then queried by BLASTP against the NCBI nonredundant protein sequence database to further support orthology relationships to well-known tunicate and vertebrate proteins. The cDNAs were cloned by RT-PCR, and the template cDNA libraries were prepared from total RNA extracted from embryos of various stages. Upstream regulatory sequences were cloned by PCR from genomic DNA (see Additional file 4). For PCRs, we used Phusion high-fidelity polymerase (New England Biolabs, Ipswich, MA) or Pfx Platinum polymerase (Thermo Fisher). Genes are named according to the proposed unified nomenclature system for tunicate genetic elements [52]. For genotyping NY1 Tyr allele, primers Moocc.genTyr1 FW and Moocc.genTyr1 REV were used. For genotyping NP1 and NP2 Tyr alleles, primers Moocc.genTyr2 FW and Moocc.genTyr2 REV were used. All the employed oligos are listed in Additional file 5.

### In situ hybridization

In situ hybridization assays were performed as previously described, with modifications [53–55]. Embryos were fixed in MEM-3.2 to 4% paraformaldehyde buffers for at least 2 h. Pre-hybridization proteinase K concentrations ranged from 0.25 (110 cell stage) to 1 (tailbud) and 5 μg/ml (larvae) for *Molgula* spp. embryos. The *Ciona robusta Tyr* and *Tyrp.a* probe was synthesized from template plasmid from the Gene Collection Release 1 [56].

Probe for fluorescent mRNA in situ hybridization of *M. occulta* Tyr was prepared from genomic clone NY1, since this gene is not transcriptionally active, and thus, no cDNA could be cloned. Probes for fluorescent mRNA in situ hybridization of *M. occulta Neurogenin* and *Onecut* were prepared from cDNA (Additional file 4). Double- and single-color fluorescent in situ hybridization was performed as previously described [53, 55].

### Sample collection and electroporation of plasmid DNA into embryos

Gravid adults of *M. oculata* and *M. occulta* were collected at the Station Biologique in Roscoff, France, during August (the only time of year when gravid animals can be found). Gonads were dissected to obtain gametes from both species. Gametes were fertilized with conspecific sperm, and embryos were allowed to develop to the appropriate stage and were then dechorionated as described previously [36]. *M. occidentalis* gravid adults were obtained from Gulf Specimen Marine Lab. Animal handling and electroporation were carried out as described in Stolfi et al. [36]. *C. robusta* (*intestinalis* ‘Type A’) adults were collected in San Diego, CA (M-Rep), and in the gulf of Taranto, Italy. Electroporation has been carried out as described [57].

## Additional files

**Additional file 1.** Figure A: Whole-mount in situ hybridization of Moocul.Tyr in *Molgula oculata* showing gene expression in one pigment cell. Table: RNAseq read counts from interspecific (*M. occulta* × *M. oculata*) embryos mapped to putative parental alleles, showing expression of *M. oculata* allele but not *M. occulta*.

**Additional file 2.** Inverted transposable elements in *M. occulta Tyrosinase* alleles NP1 and NY1.

**Additional file 3.** Database of protein sequences used in phylogenetic survey in Fig. 1, obtained from public databases (NCBI, Ensembl, Aniseed).

**Additional file 4.** Database of cloned sequences that have been used for in situ hybridizations and electroporation experiments.

**Additional file 5.** Database of entire oligo set employed for cloning DNA used in our work.
