# Supplementary material for: Evolutionary loss of melanogenesis in the tunicate Molgula occulta
Source: EvoDevo. 2017 Jul 18;8:11. doi: 10.1186/s13227-017-0074-x (PMC5516394; doi:10.1186/s13227-017-0074-x)

Table

Tyr sequence	Length (bp)	hybrid reads mapped
**Gastrula**		
occulta (NY1)	764	1
occulta (NP1)	639	0
occulta (NP2)	230	0
oculata	2067	164
**Neurula**		
**Tyr sequence**	**Length (bp)**	**hybrid reads mapped**
occulta (NY1)	764	0
occulta (NP1)	639	0
occulta (NP2)	230	0
oculata	2067	70
**Tailbud**		
**Tyr sequence**	**Length (bp)**	**hybrid reads mapped**
occulta (NY1)	764	2
occulta (NP1)	639	0
occulta (NP2)	230	0
oculata	2067	215

Figure

Molgula oculata

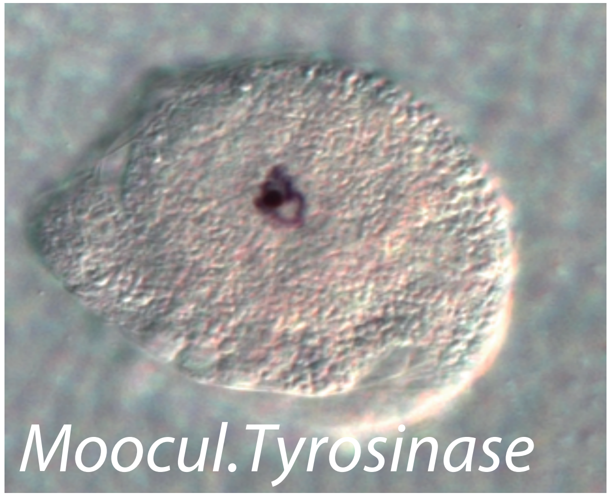

Supplement: Supplementary file 1 — Additional file 1. Figure A: Whole-mount in situ hybridization of Moocul.Tyr in Molgula oculata showing gene expression in one pigment cell. Table: RNAseq read counts from interspecific (M. occulta × M. oculata) embryos mapped to putative parental alleles, showing expression of M. oculata allele but not M. occulta. [file 13227_2017_74_MOESM1_ESM.pdf]
