# Supplementary material for: Evolutionary loss of melanogenesis in the tunicate Molgula occulta
Source: EvoDevo. 2017 Jul 18;8:11. doi: 10.1186/s13227-017-0074-x (PMC5516394; doi:10.1186/s13227-017-0074-x)
Supplement: Supplementary file 2 — Additional file 2. Inverted transposable elements in M. occulta Tyrosinase alleles NP1 and NY1. [file 13227_2017_74_MOESM2_ESM.pdf]

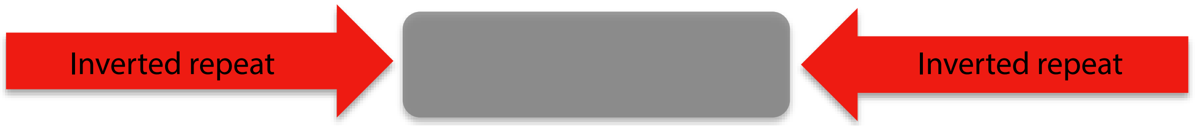


```
NP1          accacagattaagaatacgtccagatagcttacttcataggaattgtatgttctcaaacc
NY1          accacagattaagaatacgtccagatagcttacttcataggaattgtatgttctcaaacc
             ************************************************************

NP1          gttatggtccagtgaccacatgggcgaaaatcatagaaatcaaaagttacatctcatgcc
NY1          gttatggtccagtgaccacatgggcgaaaatcatagaaatcaaaagttacatctcatgcc
             ************************************************************

NP1          agaaacacttatttctgtcattatctctcacaaacttaaattaaaatttaaactgagct
NY1          agaaaaacttatttctgtcattatctctcacaaacttaaattaaaatttaaactgagct
             ***** ******************************************************

NP1          gttttattccaatcaaactgcatatacagcaacctgccagcactgggtaatcttttcaac
NY1          gttttattccaatcaaactgcatatacagcaacctgccagcactgggtaatcttttcaac
             ************************************************************

NP1          atgcagaattctttgtttacatcacgtgatgcttctccacgtggccactgagtcgcaaca
NY1          atgcagaattctttgtttacatcacgtgatgcttctccacgtggccactgagtcgcaaca
             ************************************************************

NP1          caagctcaatttcaattttaatttaagttttgtgagagataatgacagaaataagtgttt
NY1          caagctcaatttc------aatttaagttttgtgagagataatgacagaaataggtgttt
             *************      **********************************.******

NP1          ctggcatgagatgtaacttttgatttctatgattttcgcccatgtggtcactggaccgta
NY1          ctggcatgagatgtaacttttgatttctatgattttcgcccatgtggtcactggaccgta
             ************************************************************

NP1          acggtttgaaaacatacaattcctatgaagtaaactatctggacgtattcttaatctgtg
NY1          acggtttgaaaacatacaattcctatgaagtaaactatctggacgtattcttaatctgtg
             ************************************************************

NP1          gt
NY1          gt
             **
```
