# Supplementary material for: Evolutionary loss of melanogenesis in the tunicate Molgula occulta
Source: EvoDevo. 2017 Jul 18;8:11. doi: 10.1186/s13227-017-0074-x (PMC5516394; doi:10.1186/s13227-017-0074-x)
Supplement: Supplementary file 4 — Additional file 4. Database of cloned sequences that have been used for in situ hybridizations and electroporation experiments. [file 13227_2017_74_MOESM4_ESM.docx]

**- Regions used to prepare riboprobes for *in situ* hybridizations**

**>Moocul.Tyr_Moocul.CG.ELv1_2.S112652.g125 (703 bp)** TCTTCGACTACAAACAAAGGTTTAATCAGTGAATTATCGCAGTTTGCACGTTGGGAAGTCGTTTGCACTGCGGGTAACGATTACATCATCAATGGACAACAATGCACGGCGGCAGGCGAAGGACCATTATTACGAAATCCTGGAAACTTTGACGAATCGCAAACCTCAGGAATTCCAACAACAGAAGATGTTGCATCCAGCCTCCTTCTTCCTTTATACGACACACCAAATTTTGGCTCCAATGCTGATTTCAGCTTCCGAAATATTATGGAAGGGTTCGGAGATCCTTCTTCTGGCAATTCTATTGATGGTTTCAGCACGATGCACAATGCTGTTCATTTATACATGAATGGAACAATGTCAGAAGTTGGTTCCTCGGCAAACGATCCCATATTCATCCTCCACCATGCTTTTATCGACAGTTTGTATGAACAGTGGCTTCAGATTCAACAAAACAATATTTTACCCAAAGAATTGGAATTGGCCTCAGATAGAGAAATACAAAACGGTCATCGTTTGGAGGATTTCATGGTGCCCTTCTTTCCATTGGTTCGAAACTTAGAAGGTTTTGTGCGATCGGAGGATTTGGGATACGTATACGAATACTTAAATGAAAGTATGAAAGATGTGGACTGCAGTGGTGAAGAAACTGAGTCTAACGCGAATGAAATCGAGGGAGATTTCAAGGTAATTGCAACAGCTG

**>Mooccu.Tyrpa_S367316:1500..2135 (+ strand) (478 bp)**

TTGCCAGGGAACGAATGTGATGTTTGTACCGATGAACTTCTATGGAGGAAGAAACAACGAAGATCAAAATCCAAATTCTCAGTCCAAATTCTCGCTTTGCTGACTGGGATGTTGTGTGTTTATAAGTCTTCAATGGTTTGATGACAATGCGAAATTATGCAACGGAACAGCTGAGGGTAACGTTGCACTACACGCTTTTGCAAACGTGTTTTGATGAAATCGTTCAGGTCCCATTTTCCAAAATCCCGGAGGAAACGTGGATCGTCCGGAAGTTAAGAAACTTCCCGAACCTGGAGATGTCGAGGGATGTTTAAATGTTGCATCGTGCGACAGTGATCCATACTTCAGCACATCAGACAACAGCTTCAGATTGAAATGCATTGGAAGGATATGCAAAAGTTGATGGCGAATTTGCTGAAGGAGCACGAACGCTTCATAATTCGGCTCATTTGTTCTTGAACGGAACAGGAGGACAAAC

**>Mooccu.Tyr(NY-1)**

CGACCAATCCAAGAGGATAACAAGTGAAACATACATCACCCTTACTATACTTCATACAATCGGATTCTTACAGAAATCAAACCAGAATTCCGCAACGTTTCCATTTATGATTTATTTGCGTGGATGGATTAAGAATACGTCCAGATAGTTTACTTCATAGGAATTGTATGTTTTCAAACCGTTACGGTCCAGTGACCACATGGGCGAAAATCATAGAAATCAAAAGTTACATCTCATGCCAGAAACACCTATTTCTGTCATTATCTCTCACAAAACTTAAATTGAAATTGAGCTTGTGTTGCGACTCAGTGGCCACGTGGAGAAGCATCACGTGATGTAAACAAAGAATTCTGCATGTTGAAAAGATTACCCAGTGCTGGCAGGTTGCTGTATATGCAGTTTGATTGGAATAAAACAGCTCAGTTTAAATTTTAATTTAAGTTTTGTGAGAGATAATGACAGAAATAAGTTTTTCTGGCATGAGATGTAACTTTTGATTTCTATGATTTTCGCCCATGTGGTCACTGGACCATAACGGTTTGAGAACATACAATTCCTATGAAGTAAGCTATCTGGACGTATTCTTAATCTGTGGTAATAGTGTGTGGTATTACTCTTCCAAGAGACAATCTCGAGTTTTCAGATGAGGATTTGGATGACTTCATGACAACAAAAAATCAACTTCCGACAAAGGAAATGCTATCATAATTCCCCACAAAGAAATCTCCAAAATTGGTGATAGAGCCGATGCAGATTACGCATAT

**>Mooccu.Neurogenin**

AGCAGTTATGGAGGATGAGAAGAAGAATGAAGATTCTAAAAACAAGAAAAAGCGAAAGCGCGAAAGAAGTCCGGAATCAACGGTTATCCTGAAGAAGATTCGTCGTGGAAAGGCAAATGACAGAGAACGAAATCGCATGCATGGACTAAACGATGCGTTAGAAAATTTAAGGTGAGATTTGCAAATTTTTTTTTTGTTTTTTCGTTATTTTAATGCTATGTTTTTAGACGAGTTTTACCGACATATCCCGACGAAACAAAACTGACGAAGATCGAAACTTTGCGTTTTGCATACAATTACATTTGGTGTTTGAGTGAAATGATTAAAGGCAGTGAAAACGATCCAAACGCAGCAGGTAAAAATCTCATTAATTATCATTAGTAATTAACCGCTCTTGGATACCATGTTCTGTTATTATAACGTCTGCTTACACAGATTTGGCACAAGCGGCCTTCAACATGCAGCAAATGCCATCGTTCAGTGAAAACATCGTCTCCTCGACAACTTTGCATCCCATCGACGCCAATTCGCCTCCGATGATGCCGTACAATGAGTACCAACAACAGACCACATTCTCCGCCGACCAGCGACCGACTTACGGAAGTTTCGGAGGTGATTCTCGAATACACGGAGCTCATCTGCAGCAGTCATTGGTTGCAAATGAAATGGCAAATTGTCTTGATGAAATAGAAGATGTTTCACAACCATTCATCACACCATACGATCAACGACATACAAGTAGTGACGTCGGACAGCAAGGGATGGTTTATGGCAACGAAATGGCAACGTATCAATCCGCTACAACAATTAACAACAATAATAGCAACAACAATAACAACAACAACACCACTGCATTCACAAATCTAAATTCAATTGGATACCGTCGAAATGCAAAGGAAATTTCACTGCAAGGTTTAAATCAAGTGTCCCA

**>Mooccu.Onecut**

ATATGACGACGGCTCCTGTCCTGACAGATTGCGTGATGGCTACCAAAGGCAATCACAAGTAAACATGTTTCCCAACCAGACATTTCTCAATAATGGAGGATTTTCAGGCGATTTAGGAAACATTCAGAACACCTCAGCTGCTGATAGCTACGCAACCCTTCAAAATGACCAACAAGACCCTGGGTTAAGTTACGCGACCCTGACCCCACTCCAATCTTTACCGATAACTTCATCTAGTGGTGATAAATTTGTCCCTGTGCCCGTCAGTTCAAACTTCCCACTTGGTAACCCTCCGGATTCCATTGATTTAAATGGGAATTATCAAAAAATGACTGGAATGGGTCAAAGTCTTCCCCCTCTTTCTAACAGCATGTTATTAAATGGTCTTCCCACTGCCACTGATAGCGTTCACGCGCCAACGACGAGCCACGTTTCACATCAATCAGACGAAATCCCATATACCGCATCAGTGATGCATCTTCCGCAATACCCTCGATCGCCTGGTAGTTTTACCGGAACGAATCCTTACGATGCCCGAGTTTTTGACGCTGTCTCCGACACTTTTACTAACCCGATGTTCCCAGGACGGACCACAGGTTTTCCAACACCTAGCATACACAACACACGCTCGCCCATCAACACTCGCGTTAACAACAGAGGACCCAGGGGATCACCAGTCAATCTTAATAATGGGAACCAACGTCAACAAAACACTGAGGAGGTTAACACAAAAGAAGTTGCGGCGAAAATTACACAAGAACTTAAACGTTACAGCATACCACAAGCAATATTTGCACAAAGAGTTTTGTGTCGAAGCCAGGGAACACTTTCGGATCTTCTCCGCAACCCAAAACCTTGGTCCAAGCTAAAGAGTGGTCGGGAAACGTTTAGAAGAATGTGGAAGTGGCTACAAGAACCGGAATTTCAACGAATGTCGGCACTTCGACTA

**- *Cis*-regulatory region tested via transgenesis in *Molgula occidentalis* and *Ciona robusta***

**>Moocul.pTyr>GFP_S112652:5238..6629 (+ strand) (1392 bp)** TGGACAGGATATAATAGAAGCCAGAAACTGGAAACTGCGTTCAGAAGTTCAGCAGTCAGAGTTGAATGCAGAATGCAAAGCGCAGTCGATCGTCGATAACTCCCCGTCGATCTAGCACCGAATCGCGCCGAATAAGATTTTTACCCATAGAAATCAATTTGATTTGACATCGAATCGAAGGTTGCTCTTATGCCGAAGCTTATATGCACTGTTTTTTGTAATGCTAGAAATCCAATCAGTCAGGAAAAGCTCATTTTTTTCTGAATTAAACCTGCGTATTCGTAAGTCACGTGATTTAAAATAATTGTTCTTAAATAACTAACATTATTTTGTAACAATGGTAATTTATTATATAAACCACATTTTGGGGCTGAAAAAAATACTCAAGTTCACAAATCTTCTTCCTGCTTTACCATGAGTCGGTCGCAAACATTTTCTGCTTAAAAAATTTCCTTGTGAATTATCAGGAAACCGTGATTACTTATCCGTTAGCTTGTGATAGAGAGCTAAGTAACAATATTCCGTATAATCGCATAGTGCGTATAGTGCAGGGCCTAGAGTCGATTATGTAAAATTTTATTTAATAATATAGGCTACTTATATATATTCTTTAATAGCTTAAATAATCAAGTTAGGCACTTTTAAAACGATAAAGCAAAACAAATTGTGCAAAATGTGCCTAACTTTATATAAAAAAAAACTACGGTAGTTAAAATTTATGATAAAAGTGCCCAACTCACTACAAAAGAATGCTATATCTTTGACATTTGAGATGGCATTATTACCATTCTCCTTCTATCATCTTCAGGTGGACACCAGGATACTGGGGAACACATCAGTCGATCAGGTATGATTTACTAGTGATTTTATTACCAAAAACTCACACCGAAAATTTTAGTTAGGCAGTTTTTGCTTTAACTTTGCGAAACTTTCCGATTTTGTTGTTGTAGGCCTATTTTACATTGTTTGTGTGACTTGCGGTTTTTTTTTTACATTTGGGGTTTGTACCTTAATTCGCCTTTAGGTCAGCACGAATTATAACATTATTTTGCTGTTATTTATTGTCGTGACAGTCTTGCCATATTTTTTATTCTAAGTTTTGTACAGCGTCCTGGTTATTTTGTATTAAGTAATCAAAATAAATTCAATTAAATTCAAATTCACAAACTTGCATTAGATGCTCATGATGTCACGTGAGGACTTGCGGGACACATCCAATTAATTAATGACTGGGGATATAGCCTATGCACTTCCTATTAATTGAAAATCCTATTGAAAAATTGGTCTTCCTTATCAACTGAAATTACTTTAATAAACCGTTTCCCGCAACTTGGTATAAAAGTTGGCGTTCTGCTTGTGAAATATTATCATTTTACTTCATCAACAGCAAAG
